# Supplementary material for: Resting-state Alpha Reactivity Is Reduced in Parkinson’s Disease and Associated With Gait Variability
Source: Neurorehabil Neural Repair. 2025 Jun 24;39(9):742–51. doi: 10.1177/15459683251347631 (PMC12405644; doi:10.1177/15459683251347631)
Supplement: sj-docx-2-nnr-10.1177_15459683251347631 – Supplemental material for Resting-state Alpha Reactivity Is Reduced in Parkinson’s Disease and Associated With Gait Variability [file sj-docx-2-nnr-10.1177_15459683251347631.docx]

**Supplementary Table 2.** EEG alpha reactivity (mean and standard deviation) as analyzed separately for O1 and O2 electrodes for people with PD and healthy controls.

| **Electrode (alpha reactivity)** | **Parkinson** | **Control** | **p-value** |
| --- | --- | --- | --- |
| O1 / least affected side | 0.35 (0.36) | 0.57 (0.26) | 0.046* |
| O2 / most affected side | 0.31 (0.32) | 0.51 (0.37) | 0.025* |

*significant difference between groups.
