# Supplementary material for: Resting-state Alpha Reactivity Is Reduced in Parkinson’s Disease and Associated With Gait Variability
Source: Neurorehabil Neural Repair. 2025 Jun 24;39(9):742–51. doi: 10.1177/15459683251347631 (PMC12405644; doi:10.1177/15459683251347631)
Supplement: sj-docx-3-nnr-10.1177_15459683251347631 – Supplemental material for Resting-state Alpha Reactivity Is Reduced in Parkinson’s Disease and Associated With Gait Variability [file sj-docx-3-nnr-10.1177_15459683251347631.docx]

**Supplementary Table 3.** Spearman correlation coefficient and p-values for correlations between alpha reactivity and gait measures or clinical scales in people with PD.

| **Outcome measures** | **Parkinson (n=20)** |
| --- | --- |
| Step velocity | rho = 0.117, p = 0.622 |
| Step length | rho = 0.161, p = 0.498 |
| Step time | rho = 0.014, p = 0.952 |
| Swing time | rho = 0.271, p = 0.248 |
| Stance time | rho = -0.056, p = 0.816 |
| Step velocity variability | rho = -0.435, p = 0.056 |
| Step length variability | rho = -0.260, p = 0.268 |
| Step time variability | rho = -0.357, p = 0.122 |
| Swing time variability | rho = -0.544, p = 0.013* |
| Stance time variability | rho = -0.638, p = 0.002* |
| Mini Mental | rho = 0.536, p = 0.015* |
| Hoehn & Yahr stage | rho = 0.289, p = 0.075 |
| UPDRS-I | rho = 0.125, p = 0.599 |
| UPDRS-II | rho = -0.062, p = 0.794 |
| UPDRS-III | rho = -0.102, p = 0.669 |

* Significant correlation
